# An Autonomous Navigation Algorithm for High Orbit Satellite Using Star Sensor and Ultraviolet Earth Sensor

**DOI:** 10.1155/2013/237189

**Published:** 2013-10-22

**Authors:** Li Baohua, Lai Wenjie, Chen Yun, Liu Zongming

**Affiliations:** ^1^Space Control and Inertial Technology Research Center, Harbin Institute of Technology, Harbin 150001, China; ^2^Shanghai Aerospace Control and Engineering Institute, Shanghai 200233, China

## Abstract

An autonomous navigation algorithm using the sensor that integrated the star sensor (FOV1) and ultraviolet earth sensor (FOV2) is presented. The star images are sampled by FOV1, and the ultraviolet earth images are sampled by the FOV2. The star identification algorithm and star tracking algorithm are executed at FOV1. Then, the optical axis direction of FOV1 at J2000.0 coordinate system is calculated. The ultraviolet image of earth is sampled by FOV2. The center vector of earth at FOV2 coordinate system is calculated with the coordinates of ultraviolet earth. The autonomous navigation data of satellite are calculated by integrated sensor with the optical axis direction of FOV1 and the center vector of earth from FOV2. The position accuracy of the autonomous navigation for satellite is improved from 1000 meters to 300 meters. And the velocity accuracy of the autonomous navigation for satellite is improved from 100 m/s to 20 m/s. At the same time, the period sine errors of the autonomous navigation for satellite are eliminated. The autonomous navigation for satellite with a sensor that integrated ultraviolet earth sensor and star sensor is well robust.

## 1. Introduction

An autonomous navigation system of the high orbit satellite can be less reliant on, and eventually does not depend, on the ground under the condition of system support [1]. That's to say the on-orbit real-time position and velocity of satellite are calculated by themselves with the multiform sensors and navigation algorithm. The merits of the satellite autonomous navigation system not only reduce dependence on the ground level but also improve the system ability to survive. In addition, the satellite autonomous navigation system can also effectively reduce the burden of the ground control stations, ground support to reduce costs, thereby reducing the whole space program development [2].

A star sensor is a camera device which measures the direction of a star in the spacecraft coordinate system. Star sensors are widely used in spacecraft attitude determination because they produce higher accuracy attitude estimates than any other existing sensor [3, 4]. But the star sensor can only output attitude angle of aircraft relative to the inertial coordinate system for aircraft control system. However, the position and velocity information must be provided for the satellite from other sensors.

Infrared earth sensor is an optical measurement instrument, which measures the earth and the sky difference of infrared radiation and accesses satellite attitude information. Infrared earth sensors are usually used for measuring the pitch angle of aircraft attitude angle and the rolling attitude angle [5, 6]. The current satellite autonomous navigation system uses star sensor and infrared earth sensor. The starlight angular distance observed information is obtained through infrared earth sensor installation matrix vector under vehicle body coordinate system to geocentric direction of projection, and it is depending on the radius of the earth. Then, the observed variable is starlight angular distance, and the radius of earth, as the combination of satellite orbit dynamics model and filtering technology, can determine the position of the vehicle information. However, due to the fact that earth's infrared radiation characteristic is not stable, the measurement accuracy of earth radius calculated with infrared earth sensor is reduced. So, the satellite navigation information accuracy is reduced because the radius of the earth precision is reduced [7, 8]. 

 Earth edge energy at ultraviolet radiation, no matter of day or at night, is adapted to the detection of ultraviolet. The variety of earth edge energy at ultraviolet radiation extreme height is small, along with the longitude and latitude and season change. So, the edge energy at ultraviolet radiation is of enough accuracy to provide a signal for the establishment of the image sensor. Therefore, the autonomous navigation for satellite is used with earth ultraviolet band and stars visible light band [9].

## 2. Navigation Principium with the Star Sensor and Ultraviolet Earth Sensor

### 2.1. The Characteristic of Ultraviolet Radiation of the Earth

**Figure 1:**
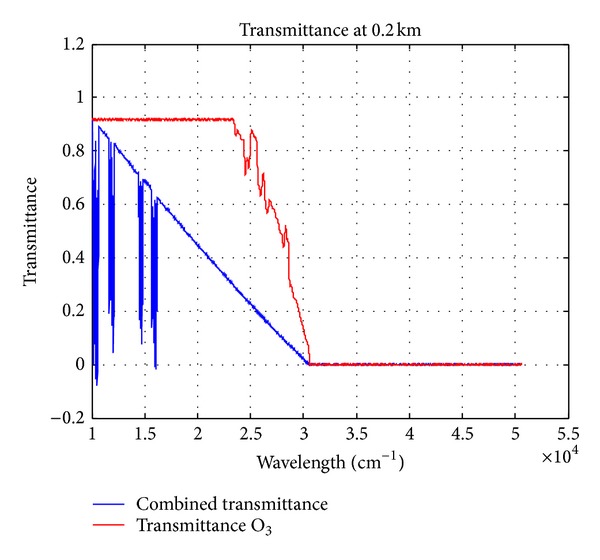
The analysis of variation between atmospheric transmittance and O_3_ transmittance.

**Figure 2:**
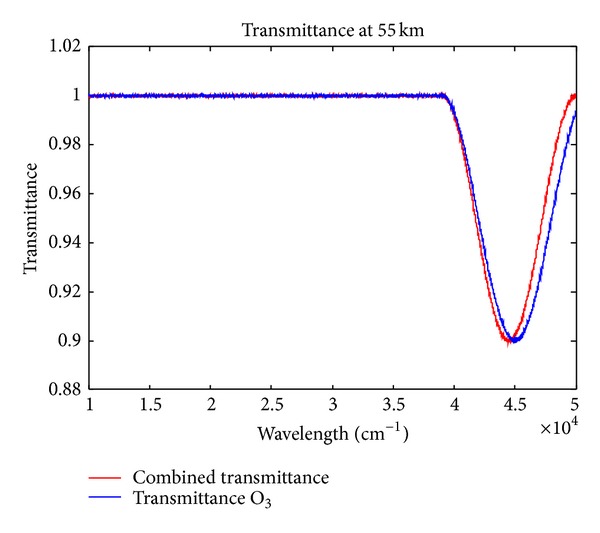
The refractivity of ultraviolet rays in the upper atmosphere.

**Figure 3:**
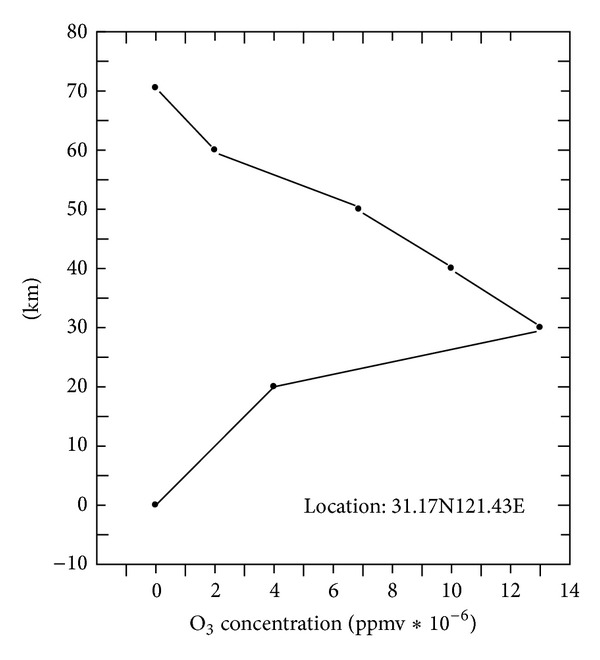
The analysis of O_3_ Concentration variation ultraviolet rays in the atmosphere.

The analysis of variation between atmospheric transmittance and ozone transmittance over 0.2 km height above sea level at ultraviolet wavelengths are shown in Figure 1. The energy radiation at ultraviolet wavelengths (200–300 nm) within the scope of atmospheric transmittance is zero. But the energy radiation at ultraviolet wavelengths (200–300 nm) is certain atmospheric transmittance. However, the energy radiation is determinate over 55 km height above sea level at ultraviolet wavelengths (200–300 nm) in Figure 2. So the energy radiation of ultraviolet can be determinate under 80 km height above sea level. The transmittance of ultraviolet wavelengths is almost 100% above 80 km height above sea level from Figure 3. So it's feasible that the autonomous navigation of satellite is used with ultraviolet earth sensors, which detect the edge of the earth at ultraviolet wavelengths.

### 2.2. Navigation Principium

The autonomous navigation system is constitutive of two Field of Views (FOVs). The first FOV (FOV1) is used to observe the stars which is called star sensor, and another FOV (FOV2) is used to observe ultraviolet radiation of the earth which is called ultraviolet earth sensor. The angle between optical axis of star sensor and optical axis of ultraviolet radiation of the earth is 90°. The star identification algorithm and star tracking algorithm are executed at FOV1. And the quaternion at J2000.0 coordinate system is calculated at FOV1. Then, the optical axis direction of FOV1 at J2000.0 coordinate system is calculated. The ultraviolet image of earth is sampled by FOV2. The coordinates of ultraviolet earth is obtained from ultraviolet image of earth of FOV2. The center vector of earth at FOV2 coordinate system is calculated with the coordinates of ultraviolet earth. The observed vector is obtained according to the geometric relationships among the earth, the optical axis of FOV1, and satellite. The position vector of satellite *R* and direction vector of *ε* satellite can be used alone; also we can use a combination of them. The accuracy of the autonomous navigation for satellite can greatly be improved with the observation combining related to the observation value of ultraviolet earth sensor and observation value of star sensor.

### 2.3. Obtaining the Coordinates of Ultraviolet Earth Image

Based on the ultraviolet earth properties, ultraviolet energy of the earth's surface is mainly caused by atmospheric reflect of the sun's ultraviolet light. So the ultraviolet image of earth in the FOV2 can never be a complete circular gaussian distribution when the satellites move around the earth. The ultraviolet image may be a circular image, or only images of the arc. So the coordinate of ultraviolet earth image is not directly obtained with centroiding algorithm.

If the shape of ultraviolet earth in the ultraviolet earth image is loop in Figure 5, the arbitrary three points called *P*1  (*x*1, *y*1), *P*2  (*x*2, *y*2), and *P*3  (*x*3, *y*3) are obtained from the loop. The line segment *l*12 is done with *P*1 and *P*2. The vertical line of the *l*12 called *L*12 is done. And the vertical line *L*12 is through the center point of the line segment *l*12. In the same way, the line segment *l*23 is done with *P*3 and *P*2. The vertical line of the *l*23 called *L*23 is done. And the vertical line *L*23 is through the center point of the line segment *l*23. The point of intersection (*x*0, *y*0) between the vertical line *L*12 and vertical line 23, which is the coordinates of ultraviolet earth image in the FOV2 coordinate system, is obtained. Then, the center vector of earth at FOV2 coordinate system is calculated with the point of intersection (*x*0, *y*0).

If the shape of ultraviolet earth in the ultraviolet earth image is arc in Figure 6, the endpoint coordinates of the arc called *P*1  (*x*1, *y*1) and *P*3  (*x*3, *y*3) are obtained from the arc. And the center coordinates of the arc called *P*2  (*x*2, *y*2) are obtained from the arc. The line segment *l*12 is done with *P*1 and *P*2. The vertical line of the *l*12 called *L*12 is done. And the vertical line *L*12 is through the center point of the line segment *l*12. In the same way, the line segment *l*23 is done with *P*3 and *P*2. The vertical line of the *l*23 called *L*23 is done. And the vertical line *L*23 is through the center point of the line segment *l*23. The point of intersection (*x*0, *y*0) between the vertical line *L*12 and vertical line 23, which is the coordinates of ultraviolet earth image in the FOV2 coordinate system, is obtained. Then, the center vector of earth at FOV2 coordinate system is calculated with the point of intersection (*x*0, *y*0).

## 3. Autonomous Algorithm

### 3.1. State Model of the Autonomous Algorithm 

Consider the following.

(1) dxdt=vx,  dxdty=vy,  dzdt=vz, r=x2+y2+z2,dvxdt=−μxr[1−J2(Rer)(7.5z2r2−1.5)]+ΔFx,dvydt=−μyr[1−J2(Rer)(7.5z2r2−1.5)]+ΔFy,dvzdt=−μzr[1−J2(Rer)(7.5z2r2−4.5)]+ΔFz.

Autonomous navigation algorithm state model of the satellite is the satellite orbit dynamics equation. There is a variety of forms of the satellite orbit dynamics equation. The most commonly the satellite orbit dynamics equation is used with celestial navigation system of rectangular coordinate expression of perturbation motion equation and Newton equations of motion among them.

The satellite orbit dynamic equation of the rectangular coordinate system is chosen at epoch (J2000.0) geocentric equatorial coordinate system. The autonomous navigation algorithm state model of the satellite is chosen as formula (1) [10].

That's to say

(2) $$\begin{array}{c} \dot{X}\left(t\right)=f\left(X,t\right)+w\left(t\right), \end{array}$$

where, $X={\left[\begin{array}{cccccc} x & y & z & v_{x} & v_{y} & v_{z} \end{array}\right]}^{T}$ is the state vector, *x* is the position scalar quantity of *X* axis at J2000.0 coordinate system, *y* is the position scalar quantity of *Y* axis at J2000.0 coordinate system, *z* is the position scalar quantity of *Z* axis at J2000.0 coordinate system, *v*_*x*_ is the velocity scalar quantity of *X* axis at J2000.0 coordinate system, *v*_*y*_ is the velocity scalar quantity of *Y* axis at J2000.0 coordinate system, *v*_*z*_ is the velocity scalar quantity of *Z* axis at J2000.0 coordinate system, *μ* is the earth' gravitational constant, *r* is the position vector at J2000.0 coordinate system, *J*_2_ is the coefficient of earth's gravity, Δ*F*_*x*_Δ*F*_*y*_Δ*F*_*z*_ are the disturbance factor from the moon, the pressure of the sun, and atmosphere of the earth, and *R*_*e*_ is the radius of the earth.

### 3.2. Observed Equation

**Figure 4:**
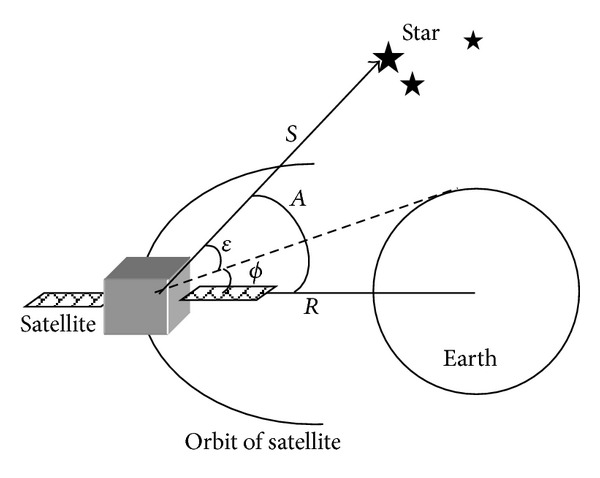
The autonomous theory with star light and ultraviolet earth.

**Figure 5:**
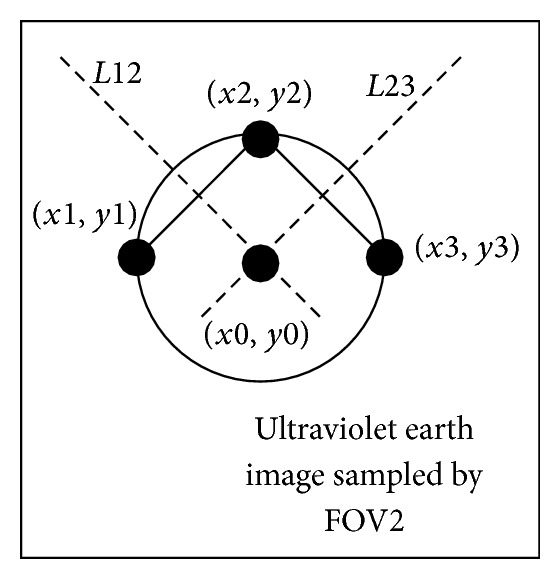
The ring image of ultraviolet earth.

**Figure 6:**
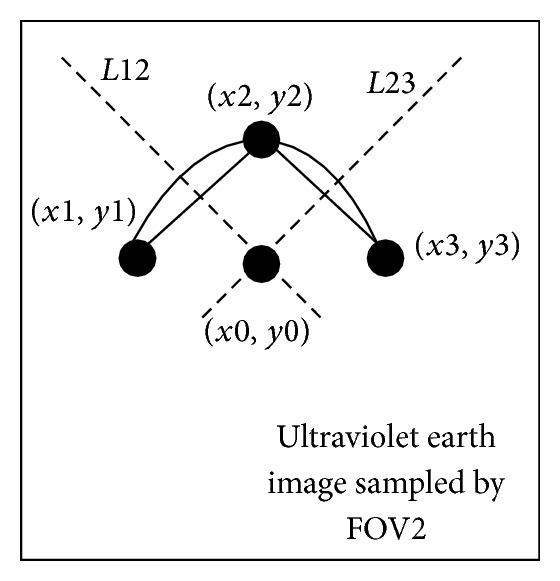
The arc image of ultraviolet earth.

The star light angle between star vector observed from the satellite and the position vector at J2000.0 is used for observed variable. According the geometric relationships from Figure 4, the star light angle *A* is calculated as follows:

(3) $$\begin{array}{c} A=\text{arc}\;\cos\left(-\frac{r\cdot s}{r}\right). \end{array}$$

So the observed equation is as follows:

(4) $$\begin{array}{c} Z\left(k\right)=A+v_{\alpha }=\text{arc}\;\cos\left(-\frac{r\cdot s}{r}\right)+v_{\alpha }, \end{array}$$

where the *r* obtained from at FOV2 is the satellite position vector at J2000.0 coordinate system, the vector *S* obtained from at FOV1 is the optical axis direction of FOV1 at J2000.0 coordinate system, and *ν*_*a*_ is the observed noise. 

### 3.3. Filter Equation

Formula (1) and formula (4) must be discrete and linearized because they are nonlinear equation, and the Extended Kalman Filter (EKF) is used for the autonomous navigation algorithm of the satellite. The discrete equation of formula (1) is as follows:

(5) $$\begin{array}{c} X\left(k+1\right)\approx X\left(k\right)+f\left(X\left(k\right),k\right)T+\omega \left(k\right). \end{array}$$

The second order Taylor series of the formula (5) at $\hat{X}(k)$ called linearization is as follows:

(6) X(k+1)≈X(k)+f(X(k),k)T+A(X(k))f(X(k),k)T22+ω(k).

where *T* is the sampling time.

The discrete equation and the linearization of formula (4) at $\hat{X}(k+1,k)$ are as follows:

(7) Z(k+1)≈B[X^(k+1,k),k] +C[X^(k+1,k),k][X(k+1)−[X^(k+1,k),k]]+ν(k).

That's to say

(8) $$\begin{array}{c} Z\left(k+1\right)\approx H\left[\hat{X}\left(k+1,k\right)\right]+\nu \left(k\right). \end{array}$$

The observed variable is the angle between the optical axis direction of FOV1 and optical axis of ultraviolet radiation of the earth FOV2. The observed matrix is as follows:

(9) $$\begin{array}{c} H=\frac{\partial Z\left(k\right)}{\partial X\left(k+1,k\right)}. \end{array}$$

The EKF filter equation based error $\Delta \hat{X}$ is as follows:

(10) $$\begin{array}{c} \Delta {\hat{X}}_{k,k-1}=\Phi_{k,k-1}\cdot \Delta {\hat{X}}_{k-1},\\ P_{k,k-1}=\Phi_{k,k-1}P_{k-1}\Phi_{k,k-1}^{T}+Q_{k-1},\\ K_{k}=P_{k,k-1}H_{k}^{T}{\left(H_{k}P_{k,k-1}H_{k}^{T}+R_{k}\right)}^{-1},\\ \Delta {\hat{X}}_{k}=\Delta {\hat{X}}_{k,k-1}+K_{k}\left(\Delta Z_{k}-H_{k}\Delta {\hat{X}}_{k,k-1}\right),\\ P_{k}=\left(I-K_{k}H_{k}\right)P_{k,k-1}{\left(I-K_{k}H_{k}\right)}^{T}+K_{k}R_{k}K_{k}^{T}, \end{array}$$

where $\Delta Z_{k}=Z_{k}-h({\hat{X}}_{k,k-1})$, *Q*_*k*_ = *E*[*ω*(*k*)*ω* ^*T*^(*k*)], and *R*_*k*_ = *E*[*ν*(*k*)*ν* ^*T*^(*k*)].

So the most merit filter equation is as follows:

(11) $$\begin{array}{c} {\hat{X}}_{k}=X_{k}+\Delta {\hat{X}}_{k}. \end{array}$$

## 4. Experiment Result and Analysis

**Figure 7:**
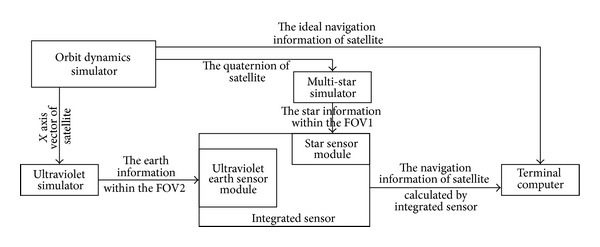
The experiment method of integrated sensor.

In order to validate the proposed autonomous navigation algorithm of high orbit satellite using star sensor and ultraviolet earth sensor, the navigation accuracy is tested in comparison with the star sensor and Infrared earth sensor. All the experiments are done with star sensor whose FOV is 14° × 14°, whose size of CCD is 1024 × 1024 pixels of 13.2 × 13.2 *μ*m pixel size, and whose update rate up is 10 Hz and the ultraviolet earth sensor whose FOV is 20° × 20°, whose size is 2048 × 2048 pixels of 5.5 × 5.5 *μ*m pixel size, and whose update rate up is 8 Hz (Figure 7). The autonomous navigation algorithm of high orbit satellite using star sensor and ultraviolet earth sensor is used for the earth synchronous orbit satellite. The orbit period of the earth synchronous orbit satellite is 24 hours. The feasibility, robustness, and navigation accuracy of the autonomous navigation algorithm of high orbit satellite using star sensor and ultraviolet earth sensor are validated under the sun in different positions. In order to reduce the amount of data sent to the PC, the sampling period of the data is 15 seconds. However, the control period of the autonomous navigation algorithm of high orbit satellite using star sensor and ultraviolet earth sensor is 200 milliseconds.

**Figure 8:**
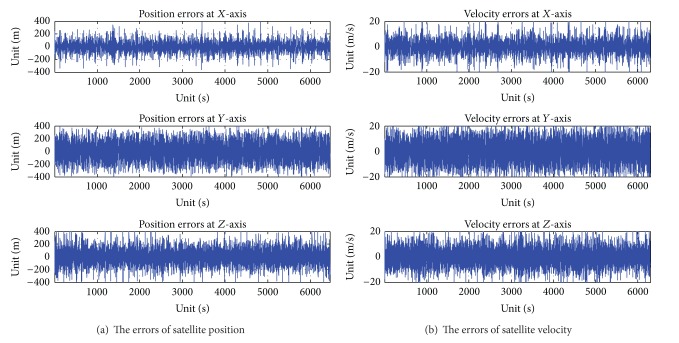
The autonomous result with a sensor that integrated ultraviolet earth sensor and star sensor.

A sensor that integrated ultraviolet earth sensor and star sensor is laid on the test bench. The star simulator is laid in front of the lens of star sensor. The star simulator is adjusted, which results in the angle between the optical axis vector of star simulator, and the optical axis vector of star sensor is 180°. The ultraviolet simulator is laid in front of the lens of ultraviolet earth sensor. The ultraviolet simulator is adjusted, which results in the angle between the optical axis vector of ultraviolet simulator, and the optical axis vector of ultraviolet earth sensor is 180°. Let the sensor that integrated ultraviolet earth sensor and star sensor put on. The RS422 is connected between the integrated ultraviolet earth sensor and star sensor and the PC. Let the star simulator and ultraviolet simulator put on. The real-time satellite orbit parameters are sent to star simulator and ultraviolet simulator with Ethernet network from orbit dynamics simulator. The star image is sampled by the star sensor of integrated sensor, and the ultraviolet image is sampled by ultraviolet earth sensor of integrated sensor. The autonomous navigation data of satellite are calculated by integrated sensor with the star image and ultraviolet image. Then, the autonomous navigation data of satellite are sent to the PC with RS422 from the integrated sensor. At the same time, the ideal navigation data of satellite to the PC are from orbit dynamics simulator. Then the errors of navigation data of satellite are calculated and saved between autonomous navigation data and ideal navigation data of satellite in the PC. The errors curves of position and velocity of satellite are showed with offline errors of navigation data in Figure 8.

**Figure 9:**
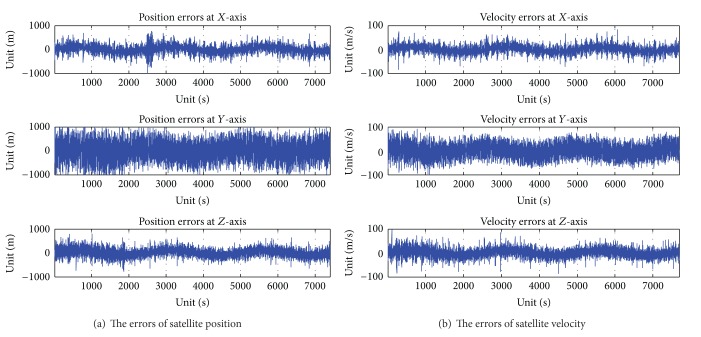
The autonomous result with a sensor that integrated infrared earth sensor and star sensor.

In order to further compare the errors accuracy of navigation using a sensor that integrated ultraviolet earth sensor and star sensor with the errors accuracy of navigation using a sensor that integrated infrared earth sensor and star sensor. The errors of navigation data of satellite are tested with a sensor that integrated infrared earth sensor and star sensor. The errors curves of position and velocity of satellite are showed with offline errors of navigation data in Figure 9. 

The autonomous navigation for satellite is calculated with a sensor that integrated infrared earth sensor and star sensor. Firstly, the infrared earth image is sampled with infrared earth sensor. Most infrared earth sensor sample the infrared earth image with CO_2_ of wavelengths 14–16 *μ*m. But the energy radiation of the infrared wavelengths is variational with day or night, longitude and latitude, and season. So the accuracy of earth radius vector is reduced with infrared earth sensor. As a result, the accuracy of autonomous navigation for satellite is reduced. The position errors of autonomous navigation for satellite are about 1000 meters, and the velocity errors of autonomous navigation for satellite are about 100 meters/second with a sensor that integrated infrared earth sensor and star sensor in Figure 9. However, earth edge energy at ultraviolet radiation, no matter at day or at night, is adapted to the detection of ultraviolet. The variety of earth edge energy at ultraviolet radiation extreme height is small, along with the longitude and latitude and season change. So the position errors of autonomous navigation for satellite are less than 300 meters, and the velocity errors of autonomous navigation for satellite are less than 20 meters/second with a sensor that integrated ultraviolet earth sensor and star sensor in Figure 8. 

## 5. Summary 

Earth edge energy at ultraviolet radiation, no matter at day or at night, is adapted to the detection of ultraviolet. The variety of earth edge energy at ultraviolet radiation extreme height is small, along with the longitude and latitude and season change. So the edge energy at ultraviolet radiation is of enough accuracy to provide a signal for the establishment of the image sensor. Therefore, an autonomous navigation algorithm for high orbit satellite using star sensor and ultraviolet earth sensor is presented. A sensor that integrated ultraviolet earth sensor (FOV2) and star sensor (FOV1) is developed. The star images are sampled by FOV1 of the integrated sensor, and the ultraviolet earth images are sampled by the FOV2 of the integrated sensor. The autonomous navigation data of satellite are calculated by integrated sensor with the star image and ultraviolet image. The star identification algorithm and star tracking algorithm are executed at FOV1, and the quaternion at J2000.0 coordinate system is calculated at FOV1. Then, the optical axis direction of FOV1 at J2000.0 coordinate system is calculated. The ultraviolet image of earth is sampled by FOV2. The coordinates of ultraviolet earth is obtained from ultraviolet image of earth of FOV2. The center vector of earth at FOV2 coordinate system is calculated with the coordinates of ultraviolet earth. The observed vector is obtained according to the geometric relationships among the earth, the optical axis of FOV1, and satellite. The position vector of satellite *R* and direction vector of *ε* satellite can be used only; also we can use a combination them. The accuracy of the autonomous navigation for satellite is doubled. And the period errors of the autonomous navigation for satellite are eliminated. So the autonomous navigation for satellite with a sensor that integrated ultraviolet earth sensor and star sensor is well robust.
